# The impact of the COVID-19 pandemic on birth, neonatal and child health and developmental outcomes in Scotland.

**DOI:** 10.23889/ijpds.v7i3.2010

**Published:** 2022-08-25

**Authors:** Louise Marryat, Bonnie Auyeung, Lydia Speyer

**Affiliations:** 1 University of Dundee; 2 University of Edinburgh; 3 University of Cambridge

## Objectives

This presentation will detail results from an initial study using linked administrative health data to explore the impact of COVID-19 lockdown measures on birth and neonatal outcomes in Scotland. It will further outline a funded longer-term follow-up study of child health and developmental outcomes using administrative health data.

## Approach

To combat the wide-spread transmission of COVID-19 Scotland imposed a nationwide lockdown. Little is known about how lockdown measures affected pregnant mothers and their offspring. Using routinely collected health data on pregnancy and birth in Scotland, the initial study compares births (N = 11220) between March and May 2020 to births in the same period in 2018 (N = 12428) to investigate the effects of lockdown measures, using descriptive statistics (Mann-Whitney U tests/Chi-squared tests). A 5-year follow-up study will track child health and developmental outcomes for the 99,000 children born in Scotland during the pandemic up to age five.

## Results

Results of the initial study indicated that mothers giving birth during the pandemic demonstrated significant differences in feeding methods on discharge (χ2(3) = 19.09, p <.001), and analgesia during labour and delivery (χ2(6) = 104.68, p <.001), and stayed in hospital for fewer days (Z = -10.90, p <.001) compared with women who gave birth in 2018. Post-hoc tests revealed that women were more likely to combine breastfeeding with formula-feeding than to exclusively breastfeed (P <.001) or exclusively formula-feed (P <.001). They were also more likely to require spinal anaesthetics compared to using no pain relief air (P =.035), gas and air (P <.001) or opioids (P < .001).

## Conclusion

Findings of the current study suggest that lockdown measures implemented in Scotland as a response to the COVID-19 pandemic had limited effects on maternal and neonatal outcomes. The CHILDS study will provide robust evidence on the longer-term impacts of the pandemic on child development, which may have long-lasting consequences for this generation.

